# Thermophysical characterisation of VO_2_ thin films hysteresis and its application in thermal rectification

**DOI:** 10.1038/s41598-019-45436-0

**Published:** 2019-06-19

**Authors:** Georges Hamaoui, Nicolas Horny, Cindy Lorena Gomez-Heredia, Jorge Andres Ramirez-Rincon, Jose Ordonez-Miranda, Corinne Champeaux, Frederic Dumas-Bouchiat, Juan Jose Alvarado-Gil, Younes Ezzahri, Karl Joulain, Mihai Chirtoc

**Affiliations:** 1 GRESPI, Multiscale Thermophysics Lab., Université de Reims Champagne-Ardenne URCA, Reims, France; 2 Institut Pprime, CNRS, Université de Poitiers, ISAE-ENSMA, F-86962 Futuroscope, Chasseneuil France; 3 Departamento de Física Aplicada, Cinvestav-Unidad Mérida, Carretera Antigua a Progreso km. 6, 97310 Mérida, Yucatán Mexico; 4 Université de Limoges, CNRS, IRCER, UMR 7315, F-87000 Limoges, France

**Keywords:** Energy science and technology, Characterization and analytical techniques, Nanoscale materials, Materials science

## Abstract

Hysteresis loops exhibited by the thermophysical properties of VO_2_ thin films deposited on either a sapphire or silicon substrate have been experimentally measured using a high frequency photothermal radiometry technique. This is achieved by directly measuring the thermal diffusivity and thermal effusivity of the VO_2_ films during their heating and cooling across their phase transitions, along with the film-substrate interface thermal boundary resistance. These thermal properties are then used to determine the thermal conductivity and volumetric heat capacity of the VO_2_ films. A 2.5 enhancement of the VO_2_ thermal conductivity is observed during the heating process, while its volumetric heat capacity does not show major changes. This sizeable thermal conductivity variation is used to model the operation of a conductive thermal diode, which exhibits a rectification factor about 30% for small temperature differences (≈70 °C) on its terminals. The obtained results grasp thus new insights on the control of heat currents.

## Introduction

The need of controlling and optimizing the performance of electronic devices with enhanced rates of operations has promoted the search for new materials with tailored physical properties^1^. Phase change materials (PCMs) characterized by the ability to significantly change their properties in a narrow interval of temperatures^2^, have attracted a lot of interest in electronics because of their ability to guide, rectify, and amplify electrical currents or thermal fluxes^3–7^. Vanadium dioxide (VO_2_) is one of these PCMs that is currently under intense research due to its reversible metal-insulator transition (MIT) occurring on a picosecond time scale^8–11^ for temperatures around 68 °C^12–14^. One of the innovative applications of VO_2_ consists in using it as one of the terminals of radiative thermal diodes (TDs)^3,4,15,16^ operating with a heat flux rectification ratio of 63%^17^, which is higher than 29% and 7% obtained for diodes based on carbon^18^ and boron nitride nanotubes^19^, respectively. In general, the ability of TDs to rectify heat fluxes can be defined by the following rectification factor^20^ *R*:

1 $$R=|{q}_{F}-{q}_{B}|/{\rm{\max }}({q}_{F},{q}_{B})$$

where *q*_*F*_ and *q*_*B*_ are the heat fluxes in the forward and backward configurations of the TDs, respectively. For the case of a radiative TDs, the values of these two heat fluxes are driven by the temperature variations of the VO_2_ emissivity, which was already measured and studied^21,22^. The sizeable contrast of the values of this property between the insulating and metallic phases of VO_2_ also drives the amplification performance of radiative thermal transistors^23–26^. By contrast, the thermal performance of conductive TDs and conductive thermal transistors based on VO_2_ are determined by its thermal conductivity (*k*) and volumetric heat capacity (*C*_*v*_)^27,28^, which are not well quantified yet, especially during the heating and cooling processes of VO_2_ around its MIT. Previous works focused on the heating process, reported that the effective thermal conductivity (*k*_*eff*_) of thin films^29,30^ and nanobeams^31^ of VO_2_ increases during the insulator-to-metal transition; while the heat capacity of VO_2_ pellets exhibits a well-defined peak for temperatures within this phase transition^32–34^. Taking into account that both the optical^13,35,36^ and electrical^37–40^ properties of VO_2_ exhibit a hysteresis behaviour (i.e., do not yield the same values during the heating and cooling processes at a given temperature within the MIT), its thermal properties are also expected to present this thermal hysteresis. However, this phenomenon has not yet been experimentally neither observed nor quantified on the thermophysical properties of VO_2_.

In this work, the hysteresis of the thermophysical properties of VO_2_ thin films deposited on either a r-sapphire or silicon substrate are measured along with the film-substrate interface thermal boundary resistance (TBR) *R*_*th*_ (Kapitza thermal resistance)^41,42^ by means of a high-frequency photothermal radiometry (PTR)^43–46^ technique. The thermal diffusivity (*a*) and thermal effusivity (*e*) were directly measured experimentally via the PTR method, and then they were used to determine both *k* and *C*_*v*_ of the thin films during their heating and cooling processes for temperatures within the VO_2_ MIT. In addition, the obtained *k* are applied to model the operation of a conductive TD and to estimate its rectification factor.

## Results

### Sample characterisation

VO_2_ thin films were deposited on r-sapphire (single crystal of Al_2_O_3_ cut through the orientation $1\bar{1}02$) and silicon (100) covered by an amorphous native oxide layer (of ∼3–5 nm thickness) substrates through pulsed laser deposition (PLD). The sample made of VO_2_ and r-sapphire will be named sample H.1, while the second one H.2. Taking into account that the sapphire substrate and VO_2_ films are semi-transparent to the visible light (pump beam of the PTR), a 30 nm-thick Ti layer was deposited on top of the VO_2_ films of both samples. For sample H.1, the deposition of Ti (with a purity of 99,99%) was made long after the VO_2_ deposition by using an electron-beam physical vapor deposition (EB-PVD, EVA 300 Alliance Concept device) at 5 10^−4^ Pa. Whereas, the deposition of the Ti layer on sample H.2 was made after the deposition of the VO_2_ film inside another secondary PLD chambers.

The crystallinity and the structural characterisation revealing the microstructure of both samples H.1 and H.2 were carried out by means of an X-ray diffraction (XRD), a field emission scanning electron microscope (FESEM) and an atomic force microscope (AFM), whose patterns/images are shown in Figs S1 and S2 of the supplementary information (SI), respectively. The crystalline structure of samples H.1 and H.2 (Fig. S1) are found strongly depended on their substrates, a r-cut-sapphire monocrystalline (H.1) in one side, and a native amorphous oxide silicon layer on a Si substrate (H.2) on the other side. The SiO_2_ amorphous layer is definitively a source of random individual grain organisation having varieties in shapes with grains enlargement in x, y and z directions, leading to a rough film (Fig. S2(f)), meanwhile the r-sapphire substrate tends to organise the grains and lead to low roughness (Fig. S2(e)). The results of the crystalline and structural characterisations of both samples are grouped in Table S1 in the SI.

### Thermophysical properties

**Figure 1:**
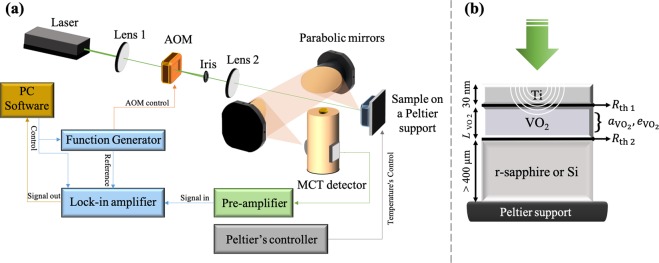
(**a**) Diagram of the used frequency domain photothermal radiometry (FD-PTR) experimental setup. (**b**) 1D heating propagation model with TBR and volumetric heat absorption in the first layer of the studied sample placed on a Peltier element with controlled temperature. The white circles in the Ti and VO_2_ layers represent the thermal waves induced by the laser heating (green arrow). *R*_th 1_ is the TBR between the Ti metallic film and the VO_2_ while *R*_th 2_ is the TBR between the VO_2_ and the substrate.

The thermophysical properties of the VO_2_ samples were measured by using their infrared emissions recorded after being heated via a laser for modulation frequencies up to 10 MHz, through a high frequency PTR experimental setup shown in Fig. 1(a) (details present in the method section).

**Figure 2:**
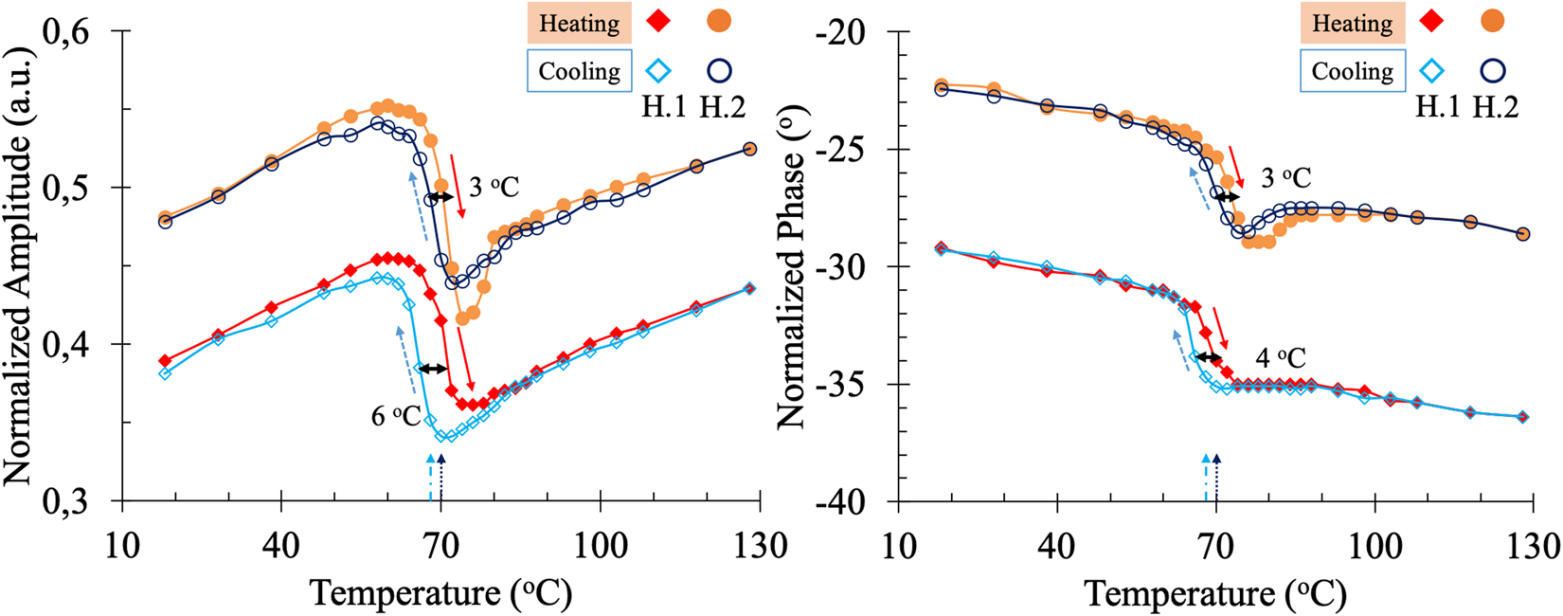
Experimental amplitude and phase of the temperature field recorded on the samples surface by using PTR at 1 MHz. The solid red and dashed blue arrows represent the heating and cooling processes of samples, respectively. Meanwhile the dashed-doted and doted arrows in the temperature axis represent the MIT temperature for sample H.1 and H.2, respectively.

The experimental amplitude and phase of the alternative temperature field recorded on the surface of both samples during their heating and cooling with temperatures from 20 °C to 130 °C, are shown in Fig. 2, for the constant modulation frequency of 1 MHz. The temperature values of the horizontal axis stand for the sample temperature resulting from the sum of the Peltier’s and the laser’s heating temperatures (an additional steady (DC) temperature raise of 8 °C has been experimentally observed and added to the sample temperature due to the laser’s heating). Note that both the amplitude and phase exhibit a clear hysteresis loop formed between the heating and cooling processes. The temperature variations of the amplitudes before and after the MIT is mainly due to the non-linearity of the PTR measurement^47^ and to the variation of the emissivity of the samples^21,22^, and therefore only the phase reflects the variation of thermophysical properties of the sample. Note that the phases of both samples change about 4° across their MIT at a transition temperature of 68 °C and 70 °C for sample H.1 and H.2, respectively. This suggests that the thermophysical properties of the VO_2_ thin films will also exhibit similar variations between the insulating and metallic phases.

In order to extract the thermophysical properties of the VO_2_ films, a frequency scan from 20 KHz to 10 MHz has been performed for various temperatures during their heating and cooling across the MIT, as shown in Fig. S3 of SI. A 1D thermal characterization of the VO_2_ films has been applied by analysing the amplitude and phase of the PTR signals recorded at each temperature, through a 1D quadrupole based model^48,49^ that takes into account a volumetric heat absorption in the first layer, as detailed in the theoretical section, below.

Afterwards, sensitivity calculations^50^ on the most significant parameters were performed. According to these sensitivities shown in Figs S4 and S5 of the SI, the amplitude and phase of the PTR signal are sensitive to the VO_2_ thermophysical properties for high modulation frequencies up to 10 MHz. The influence of the Ti coating through the Ti/VO_2_ interface TBR (*R*_th 1_) is generally negligible compared to that of the substrates through the VO_2_/substrate TBR (*R*_th 2_). The thermal diffusivity (*a*), thermal effusivity (*e*) of VO_2_ along with *R*_th 2_ were treated as fitting parameters and extracted from frequency scans of the PTR signals (example in Fig. S3) using a Gauss-Newton algorithm. The input parameters (thermophysical properties of Ti, r-sapphire, and Si) for this algorithm were taken from literature^51,52^, while *R*_th 1_ was modelled with a decay law described by P. Hopkins^53^ and E.T. Swartz & R.O. Pohl^54^. This law considers that the TBR of a metal-dielectric interface decreases as temperature increases, which is confirmed by the acoustic^42,55^ and diffusion^56^ mismatch models as well as molecular dynamics simulations^57^.

**Figure 3:**
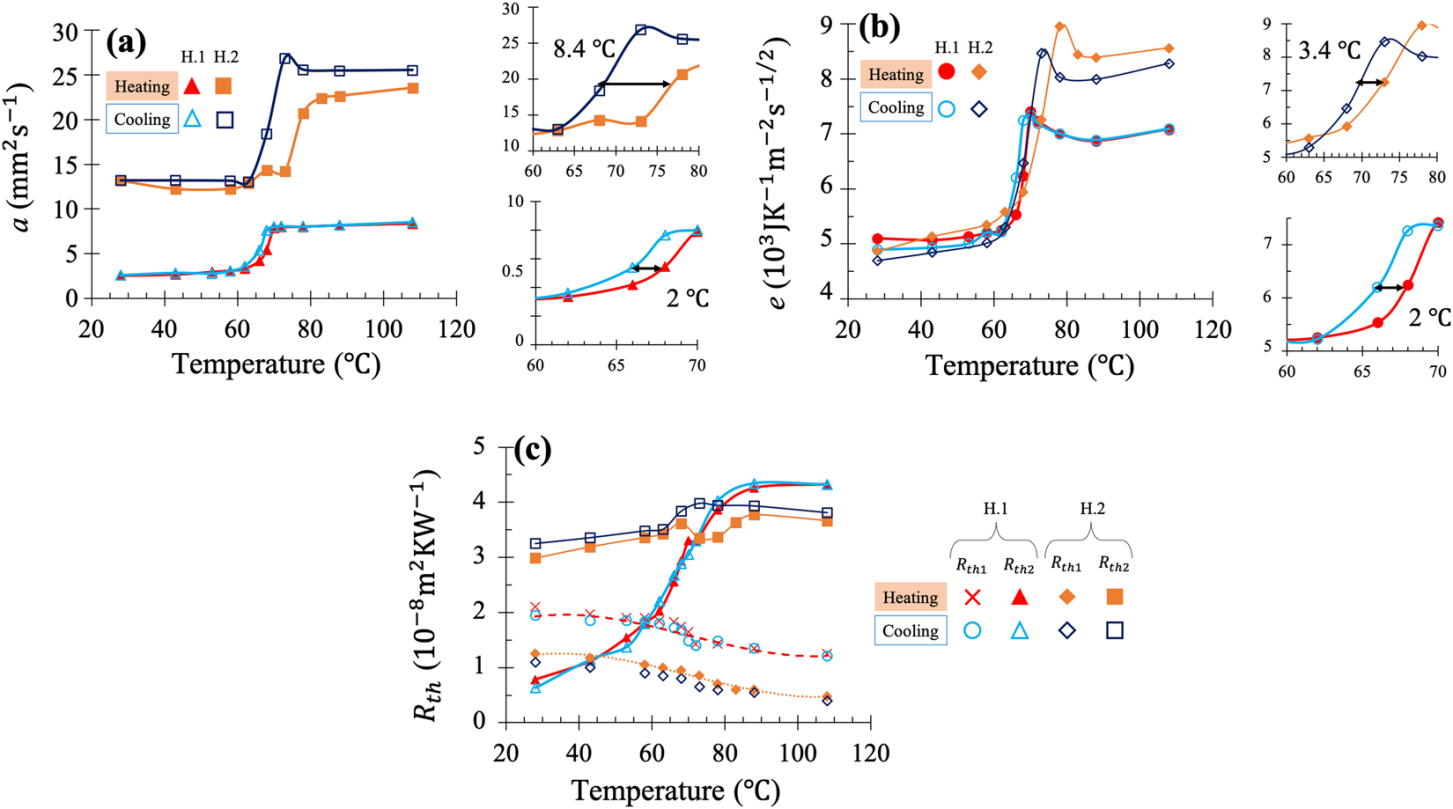
Temperature evolution of the (**a**) thermal diffusivity and (**b**) thermal effusivity of both VO_2_ films along with their (**c**) TBRs with the Ti layer and their substrates, obtained from the PTR measured signals. Dashed and dotted lines in (**c**) stand for the decay law used for *R*_th 1_. The insets at the right in (**a**) and (**b**) show closeups of the zone of the transition.

**Figure 4:**
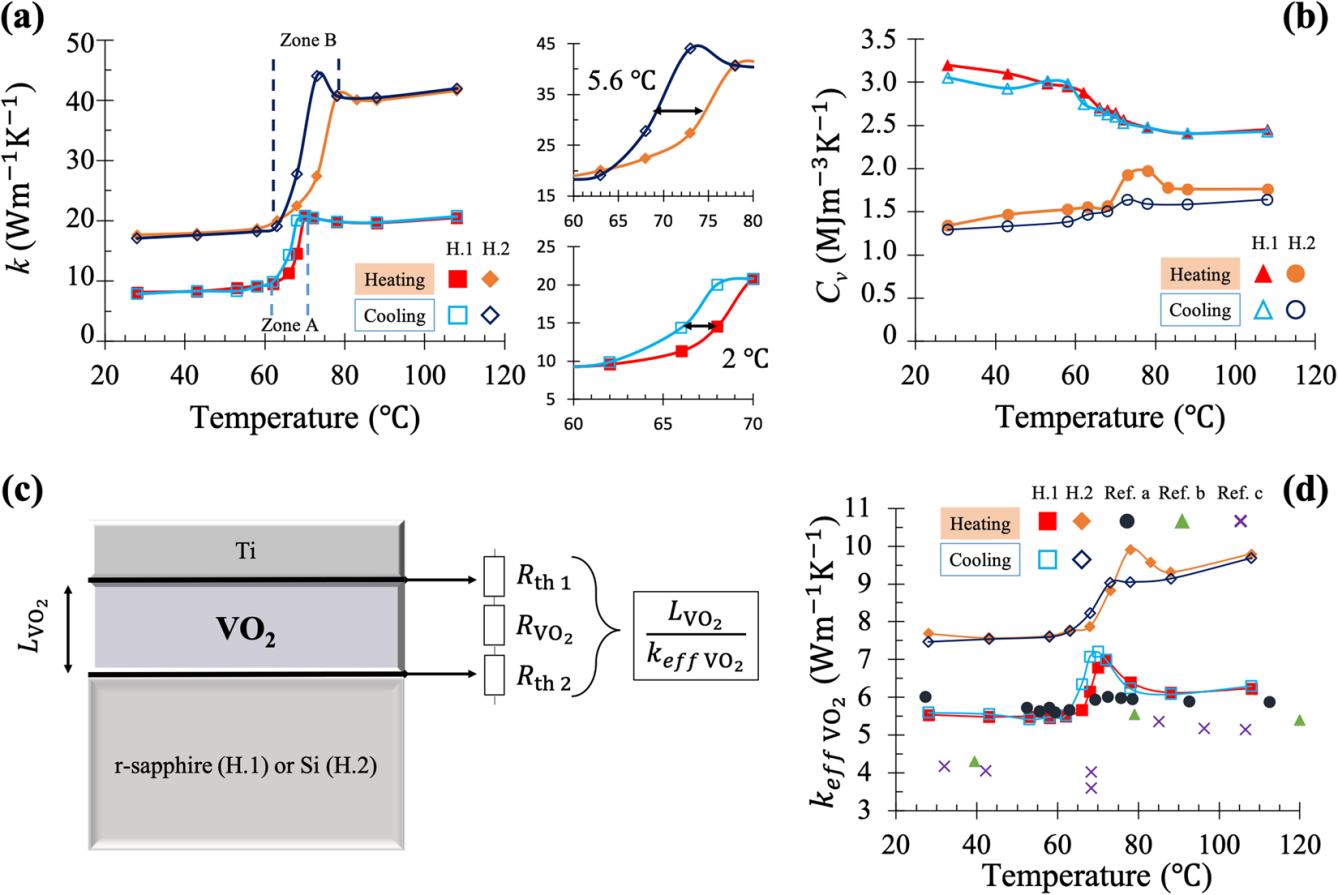
(**a**) Thermal conductivity and (**b**) volumetric heat capacity of the two VO_2_ films, as functions of their temperature. (**c**) Circuit of the two TBRs in series with the thermal resistance of the VO_2_ layer and its corresponding (**d**) effective thermal conductivity. Zone A and B in the thermal conductivity plot (**a**) represents the MIT phase transition zone of each sample. While in (**d**) Ref. a represent the results of Lee *et al*.^31^ for *k*_*eff*_of VO_2_ nanobeam; Ref. b the *k*_*eff*_ of thin VO_2_ film deposited on c-sapphire substrate (0001) by Oh *et al*.^29^; and Ref. c the *k*_*eff*_ of thin VO_2_ film deposited on quartz glass substrate by Kizuka *et al*.^30^.

The temperature evolution of the VO_2_ thermophysical properties *a* and *e* fitted for samples H.1 and H.2 are shown in Fig. 3(a,b), respectively; while the obtained values for both TBRs are displayed in Fig. 3(c). Both *k* and *C*_*v*_ were then determined through the relations: $k=e\sqrt{a}$ and ${C}_{v}=e/\sqrt{a}$, whose results are shown in Fig. 4(a,b), respectively. In addition, the effective thermal conductivity *k*_*eff*_ of the VO_2_ films with thickness *L*, taking into account the effect of both TBRs *R*_th 1_ and *R*_th 2_, has also been calculated through the well-known relation of thermal resistances in series^42^ (Fig. 4(c)): $L/{k}_{eff}=L/{k}_{{{\rm{VO}}}_{2}}+{R}_{{\rm{th}}1}+{R}_{{\rm{th}}2}$, and its values are reported in Fig. 4(d). The uncertainties over all properties were then computed using a least square algorithm^58^ which considers the uncertainties of the experimental noise and the input parameters in the model, as detailed in the SI. Their values were found equal to ±30% for *a*, ±10% for *e*, ±15% for *R*_th 2_, and ±18% for both *k* and *C*_*v*_.

## Discussion

Before discussing the thermophysical properties determined for the two VO_2_ films, the assumed decaying law for *R*_th 1_ is going to be examined. This decaying of *R*_th 1_ as temperature raises (dashed and dotted lines in Fig. 3(c)) was taken because numerous studies on metal-dielectric and metal-metal interfaces reported this behaviour associated to the electron-electron coupling, which favours the interfacial heat transport through metal-metal interfaces^45,53,59^. The difference between the absolute values of *R*_th 1_ of the two samples are not the same because the Ti layer on sample H.1 was made long after the VO_2_ one, while for H.2 the Ti was shortly deposited after the VO_2_, as pointed out in the previous section. Because of this assumption, a 15% of uncertainty has been added to *R*_th 1_ of sample H.1, through the least square algorithm used in our calculations. The impact of this error on the uncertainties of the other parameters is small, because its variation between 10-20% only changes by 2% the uncertainties of *a* and *e*.

Contrary to the Ti/VO_2_ TBR *R*_th 1_, the VO_2_/substrate TBR *R*_th 2_ of both samples increases with temperature across the MIT (Fig. 3(c)), such that *R*_th 2_ becomes higher than *R*_th 1_ in the metallic phase. This fact is reasonable because of the atomic modification of the interface after the transition degrade its quality to conduct heat through the VO_2_/substrate boundary. The significant difference between the values of *R*_th 2_ for samples H.1 and H.2 is due not only to their different substrates, but also to the presence of the very thin layer of native SiO_2_ between the VO_2_ film and its Si substrate.

Meanwhile, Fig. 3(a,b) display the influence of the MIT on *a* and *e* of both VO_2_ films. Both *a* and *e* follow a similar temperature evolution with narrow hysteresis loops, at transition temperatures around 65 and 68 °C for samples H.1 and H.2 respectively (slightly different from the temperatures of the experimental phase transition shown in Fig. 2). Interestingly, the VO_2_ films on samples H.1 and H.2 have practically the same *e* in the insulating phase (low temperature), while in the metallic phase the thermal effusivity of sample H.2 is higher than that of H.1. It is thus clear that silicon enables the growth of VO_2_ films with a higher *e* variation than r-sapphire, across the MIT. This is also the case of the VO_2_ *a* shown in Fig. 3(a) and therefore of the VO_2_ *k* displayed in Fig. 4(a).

For the VO_2_ film of sample H.1, *k* varies from 8.2 Wm^−1^K^−1^ in the insulating phase to 20.5 Wm^−1^K^−1^ in the metallic one, while for sample H.2, it goes from 16.9 to 42.2 Wm^−1^K^−1^, which represents a 2.5 enhancement of *k* through the MIT. These sizeable increases of *k* can be attributed to the complex redistribution and interactions of electrons (charge) and phonons (lattice) as well as to the spin and orbital degrees of freedom appearing during the MIT of VO_2_ which is a highly correlated electronic material^9,10,60–62^. At high temperatures, in this kind of non-Fermi liquids, the charge and heat are independently transported by distinct diffusive modes rather than carried by quasiparticles or momentum relaxation^63,64^. Thus, the increase of *k* is attributed to the presence of a new heat transport channel in the VO_2_ metallic phase associated to electronic interactions. This is explained by the fact that before the MIT (in the insulator phase) the heat conduction is dominated mainly by the lattice (phonon)^31,65^, whereas in the metallic phase both the electronic and lattice contributions are interconnected due to a redistribution and interactions of charges^30,31,65,66^. Hence, the difference between the absolute values of ${k}_{{{\rm{VO}}}_{2}}$ for samples H.1 and H.2 is mainly due to the phonon mean free path which is linked to their crystallinity driven by their substrates, as shown by the XRD patterns in Fig. S1^67,68^. Furthermore, the presented differences in the hysteresis loops width is mainly linked to the nature of the substrate and the granularity of the VO_2_, as reported in the literature^21,69,70^. In a VO_2_ single crystal, during the MIT, physical properties present strong variations (a variation of electrical resistivity of five orders of magnitude and more than 80% of optical transmission in the near IR range) in a very narrow temperature range (less than 1 K) around the transition temperature. In this paper, the two PLD VO_2_ thin films are composed by a collection of grains Fig. S2. Each grain can be a single small crystal and the global behavior of the films can be explained on one hand by each intrinsic VO_2_ single crystal properties combined on the other hand to the collective grain effect due to the microstructure of such particular thin films. Considering both samples, an approach can be based on heterogeneous nucleation^71^ and the grain size, shape, orientation and coupling would be crucial on the overall properties^72^.

The various shapes present in the VO_2_ thin film, grown on Si/SiO_2_ (sample H.2) are a consequence of the multitude of cristalline orientations linked to the SiO_2_ native amorphous surface. VO_2_ grains developed on Si/SiO_2_ substrate present various shapes and a high rugosity (root mean square deviation (AFM) Rq = 40 nm) and peak to peak value (161 nm). These disorganized grains with 3D random shape (average size x = 320 nm, y = 350 nm and z = 161 nm) are decoupled to each other, increasing the density of grain boudaries and porosity.

It has been demonstrated that VO_2_ grown on r-sapphire (sample H.1) induces a good coupling between grains (x = 200 nm, y = 650 nm and z = 28.6 nm). The single-crystal-substrate induces the growth of partially ordered grains which contributes to qualitatively improve the coupling between the substarte and the VO_2_^73^. The rugosity (Rq = 4 nm) and peak to peak values are lower than those presented for VO_2_ on SiO_2_/Si (divided respectively by 2 and 10). As a result, the density of grain boundaries, porosity and associated defects decreased leading to a stronger and sharper transition. Consequently, VO_2_ thin films synthesized on SiO_2_/Si (sample H.2) present wider thermal conductivity hysteresis equal to 5.6 °C compared to 2 °C (for sample H.1 using r-sapphire), and it has a longer phase transition (the MIT transition zone for sample H.2(zone B)) larger than the one of sample H.1 (zone A) in (Fig. 4(a)), similar to the emissivity hysteresis loops of analogous VO_2_ systems^21^.

Moreover, the values of *k*_*eff*_ for sample H.1 (Fig. 4(d)) are close to the previous ones reported in the literature^29–31^, for the heating process of VO_2_ nanobeams and thin films. This consistence of the literature values with *k*_*eff*_ and not with *k* is explained by the fact that the TBR between the VO_2_ film and its substrate was not explicitly considered in those previous works, which leads them to determine the effective thermal conductivity of their VO_2_ samples. Besides, as shown in the same figure (Fig. 4(d)), *k*_*eff*_ found by Oh *et al*.^29^ (green triangles) for thin VO_2_ films deposited on c-sapphire substrate is lower than the one obtained in this study using r-sapphire substrate (red squares). Hence, knowing that both c-(0001) and r-($1\bar{1}02$) cut-sapphire are ideal substrates for VO_2_ growth, due to their relatively small lattice mismatch, this difference in *k*_*eff*_ is linked to the orientation and nature of the VO_2_ thin films grown on either sapphire single crystals^74^.

The volumetric heat capacity *C*_*v*_ shown in Fig. 4(b) for the VO_2_ film in sample H.2 exhibits a characteristic peak within the MIT, especially during the heating process, as was observed in previous works^32–34^, while it keeps nearly constant for most temperatures outside the MIT. By contrast, the *C*_*v*_ for sample H.1 tends to decrease as the temperature increases without a clear peak for neither the heating nor the cooling processes. The significant differences on the values and temperature behaviour of the volumetric heat capacity of both VO_2_ films is associated to their differences in crystallinity (Fig. S1) and grain structure (Fig. S2), as was elucidated in previous studies^37,39,75^, as well as to a possible VO_2_ thermal dilatation (volume expansion)^75^, which is not taken into account in the used model. On the other hand, according to the sensitivity analysis (Figs S4 and S5) and the Gauss-Newton fitting procedure, it is possible to say first that the thermal effusivity results are more reliable, than the thermal diffusivity and thermal resistance ones. Secondly, the sensitivities of the PTR measurements on the VO_2_ thermophysical properties and especially on *R*_th 2_ are higher for sample H.2 (Figs S4,S5). Consequently, the VO_2_ thermophysical properties for sample H.2 are more accurate and reasonable than those for sample H.1 due to a more conductive substrate (*k*_r−sapphire_ ≈ 31.9 Wm^−1^K^−1^ and *k*_intrinsic Si_ ≈ 148 Wm^−1^K^−1^)^51,52^. Therefore, they can be taken as a reference of the thermal characterization of VO_2_ films.

Another important point is the anomalous behaviour in the form of a small peak in the *k* results of the VO_2_ (Fig. 4(a)). The peaks that results from those appearing in the *a* and *e* (Fig. 3(a,b)) are similar to the ones observed in the behaviour of the emissivity^21,22^, thermal conductivity^29,30^, electrical conductivity^31^ and optical properties^36^ of VO_2_, and are not very well understood. VO_2_, as mentioned in this discussion section, is a correlated electronic material in which the change in the comportment of electrons and phonons that occur with a change of the crystalline structure of VO_2_ during the phase transition is still not well comprehended. Further investigations are hence necessary to shed more light on this anomalous behaviour.

### Application in thermal rectification

**Figure 5:**
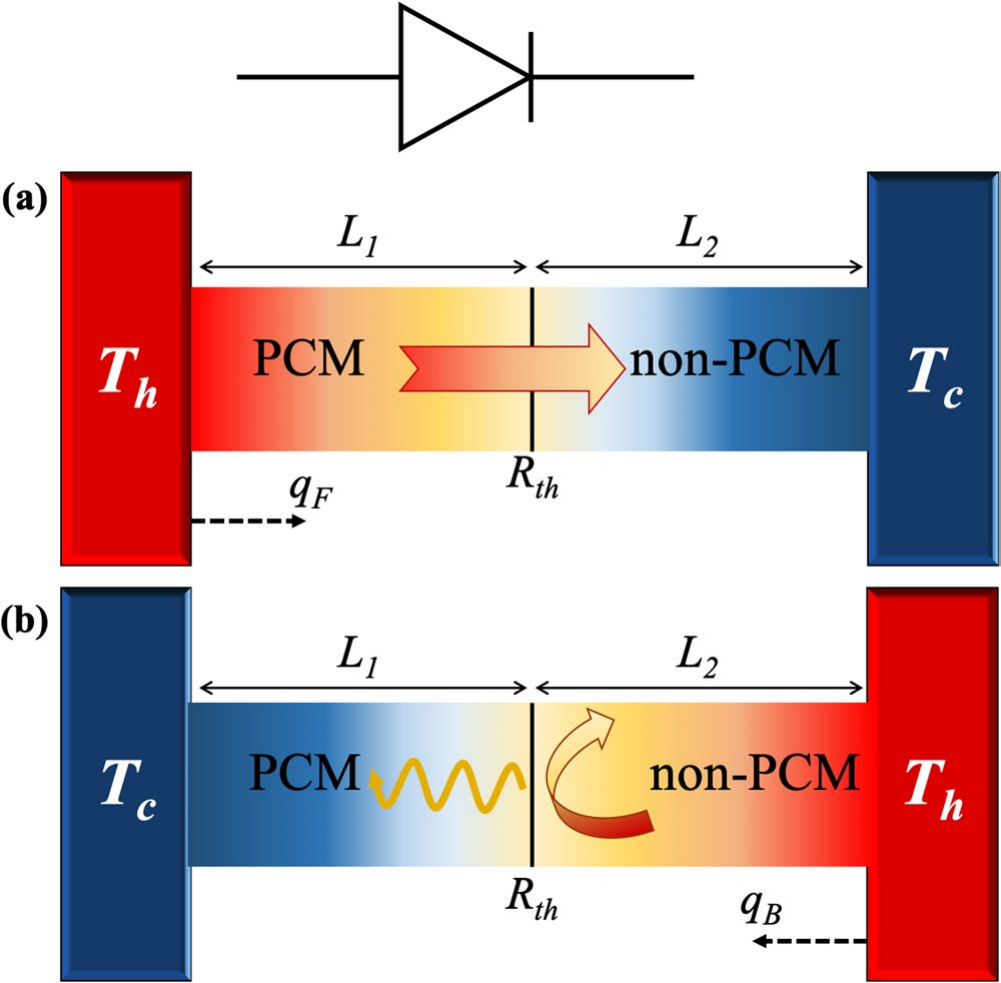
Scheme of a conductive thermal diode made up from a PCM and non-PCM in the (**a**) forward and the (**b**) backward configurations. Hot and cool thermal baths are set at temperatures *T*_*h*_ and *T*_*c*_, respectively. The combined effect of the local transitions of the VO_2_ puddles^13,37^ is taken into account through the overall transition temperature *T*_0_ in Eq. (3).

The measured temperature variations of the VO_2_ thermal conductivity ${k}_{{{\rm{VO}}}_{2}}(T)$ is now applied to assess *R* (Equation 1) of a conductive TD with VO_2_ terminal (PCM) exchanging heat by conduction with a non-PCM, as shown in Fig. 5. In the forward bias (Fig. 5(a)), the steady-state heat flux *q*_*F*_ flows from the VO_2_ terminal toward the non-PCM, while in the backward bias, an opposite direction heat flux *q*_*B*_ occurs. The values of *q*_*F*_ and *q*_*B*_ are determined by the respective thermal conductivities of the VO_2_ (${k}_{{{\rm{VO}}}_{2}}(T)$) and the non-PCM (*k*_2_), and their difference (*q*_*F*_ ≠ *q*_*B*_) is driven by the temperature dependence of $\,{k}_{{{\rm{VO}}}_{2}}(T)$ inside the VO_2_ phase transition (zones A and B in Fig. 4(a))^76^. For the sake of simplicity, we consider that inside these zones, *k*_2_ is independent of temperature and remains constant for both the forward and backward biases. The asymmetry of ${k}_{{{\rm{VO}}}_{2}}(T)$ around its transition temperature *T*_0_ allows hence to optimize the difference *q*_*F*_−*q*_*B*_ by setting *T*_*c*_ < *T*_0_ < *T*_*h*_.

Taking into account that *k* of a material is defined by the Fourier’s law of heat conduction, the experimental values of *k*_VO2_ shown in Fig. 4(a) indicates that we can apply this law to describe the heat conduction in VO_2_. According to this law and Fig. 5(a), the heat flux *q*_*F*_ is given by

2 $${q}_{F}=-\,{k}_{{{\rm{VO}}}_{2}}({T}_{1})\frac{d{T}_{1}}{dx}=-\,{k}_{2}\frac{d{T}_{2}}{dx},$$

with *T*_1_(*x*) and *T*_2_(*x*) the VO_2_ (for 0 < *x* < *L*_1_) and the non-PCM (for *L*_1_ < *x* < *L*_1_ + *L*_2_) temperatures, respectively. Based on Fig. 4(a), the measured ${k}_{{{\rm{VO}}}_{2}}$ can be modelled as follows

3 $${k}_{{{\rm{VO}}}_{2}}(T)={k}_{d}+\frac{{k}_{m}-{k}_{d}}{1+{e}^{-\beta (T-{T}_{0})}},$$

using *k*_*d*_ and *k*_*m*_ as the thermal conductivities in the insulating/dielectric (low temperature) and metallic (high temperature) phases, respectively. While *T*_0_ represents the transition temperature and *β* the phase-transition slope of ${k}_{{{\rm{VO}}}_{2}}(T)\,\,$at *T* = *T*_0_. The values of these parameters were calculated by fitting Equation (3) to the experimental data shown in Fig. 4(a) and are summarized in Table 1. The integration of Equation (2) under the usual boundary conditions of the temperature discontinuity (*T*_1_(*L*_1_) − *T*_2_(*L*_1_) = −*R*_th_*k*_2_*dT*_2_/*dx*) and heat flux continuity (−*k*_VO2_(*T*_1_)*dT*_1_/*dx* = −*k*_2_*dT*_2_/*dx*) at the interface *x* = *L*_1_, yields

4 $${q}_{F}=-\,\frac{1}{\beta \lambda {\rho }_{m}}\,\mathrm{ln}[\frac{f({T}_{h})}{f({T}_{c}+\rho {q}_{F})}],$$

where *ρ*_*m*_ = *L*_1_/*k*_*m*_, *λ* = *k*_*m*_/(*k*_*m*_ − *k*_*d*_), *f*(*T*) = [1 + *Z*(*T*)]/*Z*(*T*)^*λ*^, *Z*(*T*) = exp(−*β*(*T* − *T*_0_)), and *ρ* = *L*_2_/*k*_2_ + *R*_*th*_, with *R*_*th*_ being the TBR between the diode terminals. On the other hand, the heat flux (*q*_*B*_) in the backward configuration (Fig. 5(b)) can be determined following a similar procedure and the final result can be written as

5 $${q}_{B}=-\,\frac{1}{\beta \lambda {\rho }_{m}}\,\mathrm{ln}[\frac{f({T}_{h}-\delta T+\rho {q}_{B})}{f({T}_{c}+\delta T)}],$$

where *δT* = 2 °C (or 4.5 °C) is the hysteresis undergone by the VO2 thermal conductivity, as the terminal deposited on r-sapphire (or silicon) is cooled down from *T*_*h*_ in the forward configuration, to *T*_*c*_ in the backward one, as shown in Fig. 5. Note that the thermal resistance *L*_2_/*k*_2_ of the non-PCM influences both *q*_*F*_ and *q*_*B*_ through the total resistance *ρ*, which is symmetric in *R*_th_ and *L*_2_/*k*_2_. This fact indicates that the impact of interface TBR between the diode terminals can be neglected by choosing a non-PCM, such that *L*_2_/*k*_2_ ≫ *R*_*th*_ ~ 10^−8^m^2^KW^−1^(see Fig. 3(c)), as will be considered in this work.

**Table 1:** Materials properties of VO_2_ deposited on r-sapphire and silicon substrates.

Material	*k*_*m*_(Wm^−1^K^−1^)	*k*_*d*_(Wm^−1^K^−1^)	*β*(°C^−1^)	*T*_0_(°C)
VO_2_/Sapphire	20.5	8.2	0.4	67.5
VO_2_/Silicon	41.7	17.5	0.3	72.5

**Figure 6:**
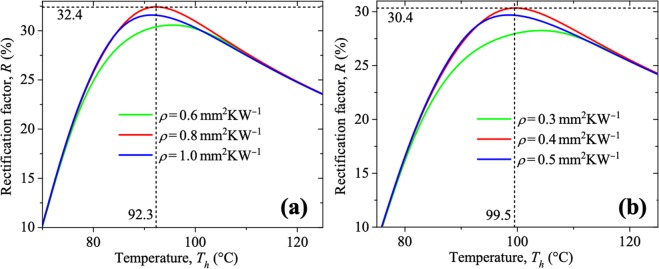
Dependence of the rectification factor on the temperature of the hot side of a conductive thermal diode with a terminal of VO_2_ deposited on a substrate of (**a**) r-sapphire and (**b**) Silicon. Calculations were done for three representative values of the thermal resistance *ρ, L*_1_ = 10 μm and *T*_*c*_ = 25 °C.

The plots in Fig. 6(a,b) represent the variation of *R* as a function of the temperature *T*_*h*_ of the hotter terminal for both conductive TD operating with terminals of VO_2_/r-sapphire as well as VO_2_/Si. Calculations were done with the numerical solutions of Equations (4) and (5) for *q*_*F*_ and *q*_*B*_, in combination with Equation (1).

One can note that the TDs operating with VO_2_/r-sapphire and VO_2_/Si reach their optimal rectification factors *R*_opt_ = 32.4% and *R*_opt_ = 30.4% at the temperatures *T*_*h*_ = 92.3 °C and *T*_*h*_ = 99.5 °C, and thermal resistances *ρ* = 0.8 mm^2^KW^−1^ and *ρ* = 0.4 mm^2^KW^−1^, respectively. Higher and lower values of *ρ* and *T*_*h*_ yield lower rectification factors. Even though both diodes have practically the same highest rectification factor, the TD operating with terminals of VO_2_/r-sapphire and a blackbody requires a terminals’ temperature difference (*T*_*h*_ − *T*_*c*_ =  67.3 °C) that is smaller than the corresponding one (*T*_*h*_ − *T*_*c*_ = 74.5 °C) for the diode composed of terminals of VO_2_/Si and a blackbody. This difference is due to the fact that the first VO_2_ sample has a smaller transition temperature *T*_0_ and faster phase transition (higher *β*) than the latter one, as reported in Table 1. Additionally, the evidence that these temperature differences between the diode terminals (*T*_*h*_ − *T*_*c*_) are larger than the equivalent phase transition zones of both samples shown in Fig. 6 is acceptable, given that the maximization (minimization) of the forward (backward) heat flux *q*_*F*_ (*q*_*B*_) associated to *R*_opt_ occurs when *T*_*h*_ (*T*_*c*_) is high (low) enough to ensure that most of the VO_2_ terminal is in its metallic (insulating) phase, as established by the Fourier’s law of heat conduction. This fact suggests that VO_2_ samples with sharper *k* slopes require smaller temperature intervals to reach the maximum *R* of a conductive TD, as was theoretically demonstrated in the literature^77^. Moreover, *R*_opt_ values determined for VO_2_/r-sapphire and VO_2_/Si are higher than the corresponding one (*R*_opt_ = 19.7%) reported in the literature for another VO_2_ sample^77^, because their thermal conductivity contrasts *k*_*m*_/*k*_*d*_ = 2.5 are larger than that (*k*_*m*_/*k*_*d*_ = 1.7) of the latter sample. Thus, it is suggested that PCMs with higher *k* deviations, within their MIT, are expected to generate a more conductive TD with higher *R* that can even exceed that obtained using graphene (26%)^78^.

The obtained outcomes lay down a novel methodology to study the thermophysical properties of VO_2_ thin films and other PCMs with potential applications in the control of heat currents. As shown in this and previous works^21,79,80^, the substrate plays a key role on the growth and thermal properties of VO_2_ films, and therefore the detailed study of its impact with a wider variety of substrate materials, film thicknesses and deposition temperatures is a pending task.

## Conclusion

In this study, experimental thermal characterisation of vanadium dioxide thin films were achieved using a PTR setup up to 10 MHz. The reported measurement of the VO_2_ thermophysical properties (${a}_{{{\rm{VO}}}_{2}}$, ${e}_{{{\rm{VO}}}_{2}}$ and *R*_th 2_) was made. The hysteresis loops of these properties have been observed and quantified by performing all measurements for both the heating and cooling processes of samples with different temperatures within their MIT. It has been shown that: i) the *a*, *e* and *k* of both VO_2_ films in their insulating phase, are lower than the corresponding ones in their metallic phase. The *k* of both VO_2_ films exhibits a substantial enhancement of 2.5 during the MIT, whereas, their *C*_*v*_ display smaller variations. ii) MIT reduces the heat transmission at the interface through the phase changing. iii) This high *k* contrast of VO_2_ allows to drive a conductive TD with a rectification factor as high as 30%, for a relatively small temperature difference between its two terminals of VO_2_ and another non-PCM. The obtained results are thus expected to guide the fabrication of VO_2_ films and other PCMs with thermal properties tailored for the control of the heat conduction in many technological applications.

## Methods

### Sample preparation

VO_2_ thin films were deposited on substrates of r-sapphire ($1\bar{1}02$) and silicon (100) wafers through a pulsed laser deposition (PLD) process. This well-adapted method for the deposition of complex multi-element materials and oxides consists in using a pulsed high-power laser beam to evaporate a small amount of matter from a solid target within a stainless-steel ultra-high vacuum chamber^21^. During some hundreds or thousands of laser pulses, the evaporated matter condensed on a substrate forms a thin film. A KrF pulsed excimer laser with wavelength *λ* = 248 nm, pulse width of 25 ns, and repetition rate ranging from 3 Hz up to 25 Hz was employed to grow VO_2_ films on both types of substrates set at the temperature of 600 °C. The oxygen pressure used for the deposition of all samples was of 2.2 Pa. The thicknesses of the VO_2_ thin films of samples H.1 and H.2 were measured by a profilometer (KLA Tencor, Alpha-Step IQ) and their values were found to be of 500 nm and 400 nm (± 20 nm), respectively.

### PTR measurements

The photothermal radiometry (PTR) technique detects the infrared signals emitted by a material after being heated up with a frequency modulated laser beam and analyses them to find the material’s thermophysical properties^43–45^. The standard PTR setup used in our experiments uses an optical excitation provided by a continuous diode-pumped solid-state laser (DPSS, Dream Lasers Technol. Co., model SDL-532-300T) with an intensity of 300 mW, visible wavelength of 532 nm and a diameter of 0.6 mm at *1/e*. Modulation frequencies up to 10 MHz were achieved by an acousto-optical modulator (AOM model AA.MTT.AR 05) placed between two lenses in their confocal plane. The laser beam spot inside the AOM driver controlled by a frequency generator (model FI5350GA) is thus minimized and the upper limit of the modulation frequency was determined by the transit time of the acoustic carrier frequency across the laser beam spot. An iris was used to select the zero-order diffracted beam. The intensity of the laser beam arriving on the sample surface is around 55% of the initial power after its passage through all the involved optics. The VO_2_ samples were mounted on a Peltier support equipped with a temperature controller (Newport temperature controller Model 6100) via a Pt100 probe. The surface temperature of the samples was measured with a small thermocouple probe (YCT RS-232 Data Logger, YC-747D Thermometer, probe diameter = 85 µm) and was found to be equal to that registered by the Peltier support. An additional steady (DC) temperature raise of 8 °C has been experimentally observed and added to the sample temperature due to the laser’s heating. The infrared (IR) radiations generated from the sample heating are collected by two parabolic off-axis Au-coated mirrors (Edmund Optics) and transferred to a liquid-nitrogen cooled HgCdTe photoconductive detector sensitive to the IR radiation wavelengths between 5 and 12 µm (Kolmar Technologies, KMPV 11-1-J1/DC) with 1 mm^2^ active area and antireflection-coated Ge window. The amplitude and phase of the detector electrical signal are then recorded and amplified through heterodyne lock-in amplifiers (Stanford Research Systems: SR844). The function generator used to modulate the AOM driver was also used as base reference of the lock-in frequency. A schematic representation of the used PTR system is shown in Fig. 1 and more details about it can be found elsewhere^45^.

### Theoretical calculations

Using such experimental setup configuration, the heating is considered to be one-dimensional. This can be explained by the relation between the laser spot size, the detector opening and the excitation source frequency. I.e., knowing that when the thermal diffusion length defined as *μ* = (*a*/*πf*)^1/2^ (where *a* and *f* are the thermal diffusivity and the laser modulation frequency respectively) in meters is smaller than the opening of the detector, the heating is considered 1D (with a laser beam spot size equal to 0.6 mm at 1/e). Consequently, at enough high frequencies, the VO_2_ layer is considered as thermally thick, because under these conditions, the penetration depth of the thermal wave (defined as *μ*) (whites circles in Fig. 1(b)) is small, in such a way that the thermal waves remain in the first two layers (Ti and VO_2_) of the samples. For that reason, a quadrupole based model^48,49^ including a volumetric heat absorption in the first layer (50 10^6^ for Ti) is used to fit the experimental data (normalized amplitude and phase simultaneously). This modelling was used to simultaneously fit the experimental data for the amplitude and phase of the PTR signal analysed by the lock-in amplifier. A sensitivity calculation^50^ is also made to check which are the parameters that can be extracted, sensitive to this experimental measurement (details in section 3 of the SI). Subsequently, these sensitivities were used to compute the total uncertainties of the extracted parameters, using a least square algorithm as explained by another study^58^. This method calculates the total error on an experimental parameter using both, the uncertainties related to the experimental noise and the input parameters (the fixed parameters in the model).

## Supplementary information

Supplemental information for: “Thermophysical characterisation of VO<sub>2</sub> thin films hysteresis and its application in thermal rectification”

## Data Availability

The datasets generated and/or analysed during the current study are available on request to the corresponding author.
