# Supplementary material for: Thermophysical characterisation of VO2 thin films hysteresis and its application in thermal rectification
Source: Sci Rep. 2019 Jun 19;9:8728. doi: 10.1038/s41598-019-45436-0 (PMC6584564; doi:10.1038/s41598-019-45436-0)
Supplement: Supplementary file 1 — Supplemental information for: “Thermophysical characterisation of VO2 thin films hysteresis and its application in thermal rectification” [file 41598_2019_45436_MOESM1_ESM.pdf]

Supplemental information for: “Thermophysical characterisation of VO_2 thin films hysteresis and its application in thermal rectification”

Georges Hamaoui[1], Nicolas Horny[1*], Cindy Lorena Gomez-Heredia[2,3], Jorge Andres Ramirez-Rincon[2,3], Jose Ordonez-Miranda[2], Corinne Champeaux[4], Frederic Dumas-Bouchiat[4], Juan Jose Alvarado-Gil[2,3], Younes Ezzahri[2], Karl Joulain[2], Mihai Chirtoc[1]

[1]GRESPI, Multiscale Thermophysics Lab., Université de Reims Champagne-Ardenne URCA, Reims, France
[2]Institut Pprime, CNRS, Université de Poitiers, ISAE-ENSMA, F-86962, Futuroscope Chasseneuil, France
[3]Departamento de Física Aplicada, Cinvestav-Unidad Mérida, Carretera Antigua a Progreso km. 6, 97310, Mérida,Yucatán, Mexico
[4]Université de Limoges, CNRS, IRCER, UMR 7315, F-87000, Limoges, France

[*]nicolas.horny@univ-reims.fr

This document contains additional information to the manuscript entitled **“Thermophysical characterisation of VO_2 thin films hysteresis and its application in thermal rectification”.** It provides supplementary details on the crystalline and structural characterisation of the samples, the sensitivity and uncertainty calculations, and on experimental data analysis in support of the manuscript.

1. Sample structural characterisation details

The crystallinity of samples H.1 and H.2 were studied by means of X-ray diffraction (XRD), whose patterns are shown in Figure S.1. Considering the relatively high intensity of the silicon peaks compared with the ones of sapphire, the diffraction analysis of sample H.2 was developed with a diffractometer Siemens D-5000 operating with a grazing incidence and $CuK_{\alpha 1}$ radiation, while that of sample H.1 was recorded with a diffractometer D-8 advance, working with a Bragg-Brentano (θ, 2θ) geometry. According to the International Centre for Diffraction Data (Card 04-003-2035)[1], both samples show a crystalline structure characteristic of the monoclinic phase of VO_2 (red lines). The VO_2 film deposited on silicon (blue line) do not have a preferential orientation and displays a principal peak in the plane (011), while the one grown on r-sapphire (black line) presents an orientation at the (200), (111) and (400) diffraction peaks, which agree with those reported in the literature[2,3]. The crystalline structure of samples H.1 and H.2 thus depends strongly on their substrates, a r-cut-sapphire monocrystalline (H.1) in one side, a native amorphous oxide silicon layer on a Si substrate (H.2) on the other side.

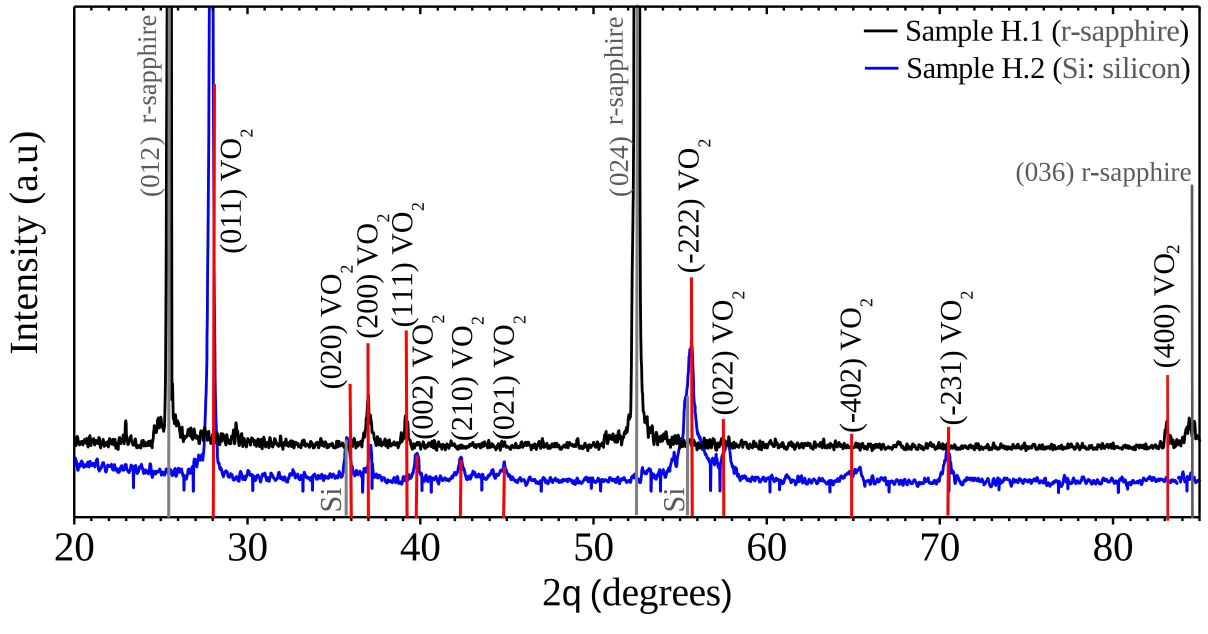

Figure S.1: Room temperature XRD patterns of samples H.1 (black line) and H.2 (blue line Grey lines stand for the principal XRD peaks of the substrates.

Furthermore, based on a field emission scanning electron microscope (FESEM JEOL 7600F) and an atomic force microscope (AFM) integrated to a confocal Raman Witec Alpha300

spectrometer, the structural characterization revealing the microstructure of both samples was also carried out.

The images obtained for sample H.1 (VO_2+sapphire) and sample H.2 (VO_2+silicon) are respectively shown in Figures S.2(a,c,e) and S.2(b,d,f) which were used to determine their average grain-size distributions of about 0.63 μm(length) × 0.2 μm(width) and 0.35 μm × 0.32 μm, respectively. The crystallite sizes where also determinded using the Scherrer's formula on the XRD patterns. The peak width of diffraction pattern, in absence of deformations due lattice strain and faulting, mainly depend on crystallite domain size (D), which can be determined by using the Scherrer´s formula[4].

$$D = \frac{K\,\lambda}{\mathrm{FHWM}\cos\theta} \tag{S.1}$$

The FHWM is obtained fitting the peak with a Gaussian function and is the full width at half-maximum (in radians) which represents the crystallite dimension in the *z* axis; where λ is the wavelenght of the Cu Kα radiation (0.15406 nm) used to obtain the XRD pattern, θ is the diffraction angle at witch the maximum is present (deg) and K is a constant which depends on the particle. If we assume that average volume particle size of VO_2 crystallite is independent of morphology, it is possible to take K=1 rad. The *x-y* and *z* dimensions of both samples (H.1, H.2) have been calculated by SEM and AFM images, respectively.

Now, using the Scherrer's formula and the main peaks of the XRD patterns, we estimate the average of crystallite size of both samples. The average crystallite size (D) of sample H.1 is higher (1.47 times) than for sample H.2, associated with the preferential orientation of the crystallite growth, similar to the results found by crystal's length (*y* axis) calculation (1.8). This confirms the good coupling among the VO_2 grains of sample H.1 due to substrate characteristics. All the crystalite results are grouped in the following table:

Table S.1: Structural properties characterisation results of both types of sample using the AFM, XRD and SEM results.

Property	Sample H.1	Sample H.2
Thickness (nm)	500	400
Root mean square deviation (AFM) Rq (nm)	~3-4	~30-40
XRD mean peak position (2θ) (deg)	25.49	27.75
FWHM (rad)	0.00207	0.00393
Average crystallite size D (XRD) by Scherrer's formula (nm)	59.49	40.37

Grain size (SEM micrographs) (nm)<br>x: width<br>y: length	x=200<br>y=630	x=320<br>y=350
Grain height (AFM imagen) (nm)	z=28.6	z=161

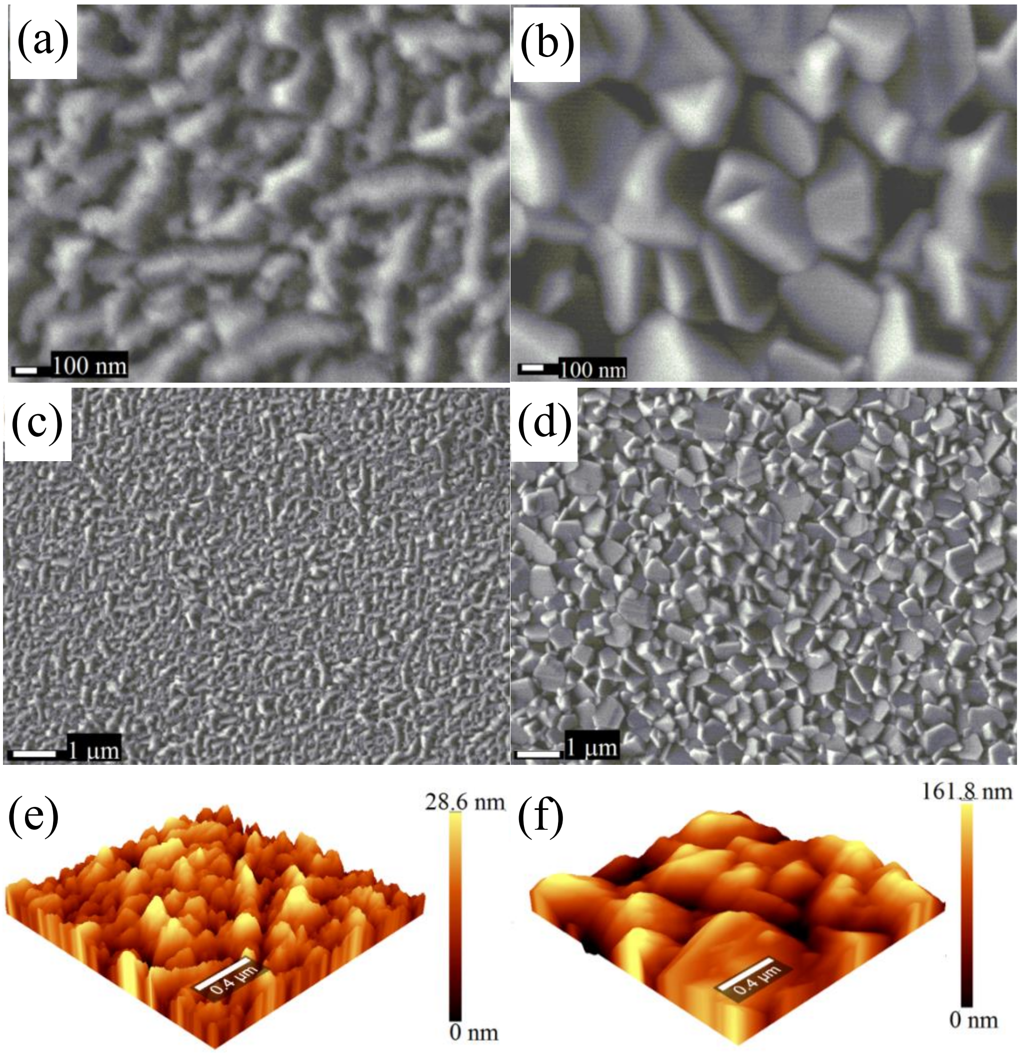

Figure S.2: FESEM and AFM images obtained for samples H.1 (a, c, e) and H.2 (b, d, f). SEM images were taken for 50.000X (up) and 10.000X (down).

From these figures, it is clear that the growth mechanisms for the two substrates are different and lead to 3D ad-atom clusters (grains) which present different organisations and shapes. Isolated grains, weakly connected with sharp, pyramidal and cubic shapes are clearly observed for sample H.2, whose varieties in shapes and disorganised disposal are due essentially to the amorphous SiO_2 layer. In contrast, grains formed in sample H.1 exhibit spherical, elongated and well interconnected grains induced by influence of the sapphire substrate, which is consistent with previously reported results[2]. The SiO_2 amorphous layer is

definitively a source of random individual grain organisation with grains enlargement in x, y and z directions, leading to a rough film (Figure S.2(f)) while the r-sapphire substrate tends to organise the grains and lead to low roughness (Figure S.2(e)).

The FESEM, AFM and XRD show huge differences between VO_2 films deposited on r-sapphire and SiO_2/Si, respectively. In this layer thickness, in the range of 400-500 nm (details present in the method section in the main text), the crucial role of the substrate is evident on both the structure and the microstructure.

2. Theoretical model

PTR experimental method uses a Gauss-Newton mathematical algorithm to obtain the selected fitting parameters. However, two types of minimization exist, one uses the amplitude and the phase of the thermal signal for the calculation, and the other one just the phase. When considering both amplitude and phase profiles, the minimization includes more experimental data in the analysis, leading to a better estimation of the fitting parameters. For that reason, in the paper, both normalized amplitude and phase profiles were taken for the PTR minimization. Figure S.3 regroup an example of the experimental data using a full frequency scans (from 20 KHz to 10 MHz) normalized to a reference sample (polished metallic alloy sample of Aluminium, Vanadium and Titanium (TA6V)) and theoretical model for two temperatures (28 and 78 °C) at the two different phases (insulator and metallic).

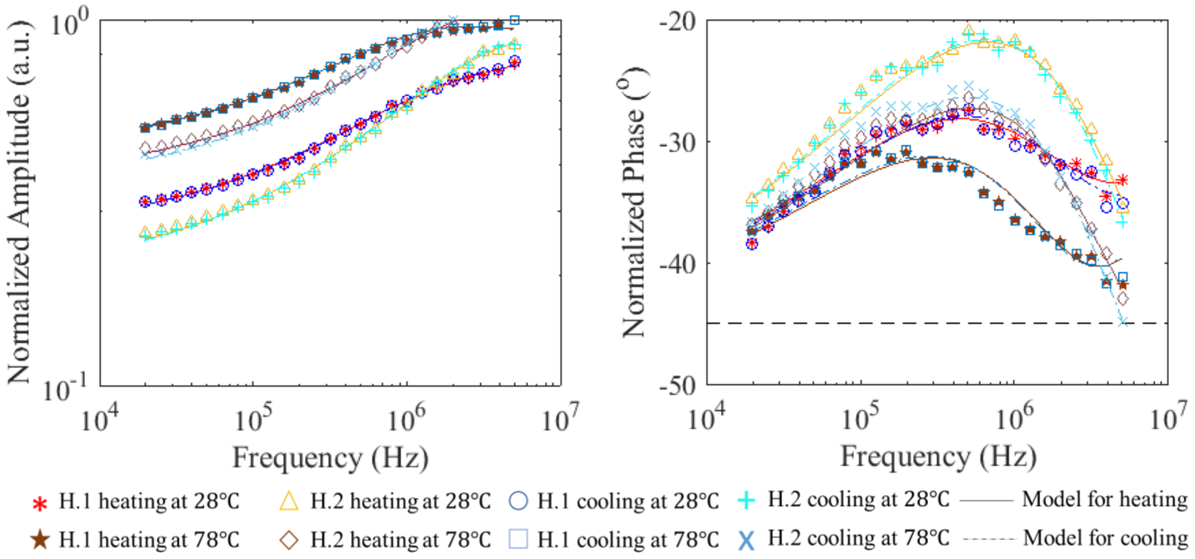

Figure S.3: Experimental and model fitting of amplitude and phase of surface temperature T_{AC} for both H.1 and H.2 samples for dielectric (28°C) and metallic (78°C) phases using PTR (Black dashed line in the phase plot at -45° is guide for eyes).

From Figure S.3, one can see that the heating and cooling plots superpose. This means that, during and after this reversible MITs, the properties of each layer regain their original status.

3. Sensitivity and uncertainty calculations

Sensitivity calculation is also made to check which are the parameters that can be extracted, sensitive to this experimental measurements using the relative sensitivities[5,6] $S_p^A = \frac{\partial \ln A}{\partial \ln p}$ and $S_p^\varphi = \frac{\partial \varphi}{\partial \ln p}$ (where S_p^A and S_p^φ are the relative sensitivities related to the effect of parameter p variation on the amplitude and phase profiles respectively). An example containing the intrinsic parameters (thicknesses, thermophysical properties of all layers and TBRs) of the studied sample is displayed in Figures S.4 and S.5.

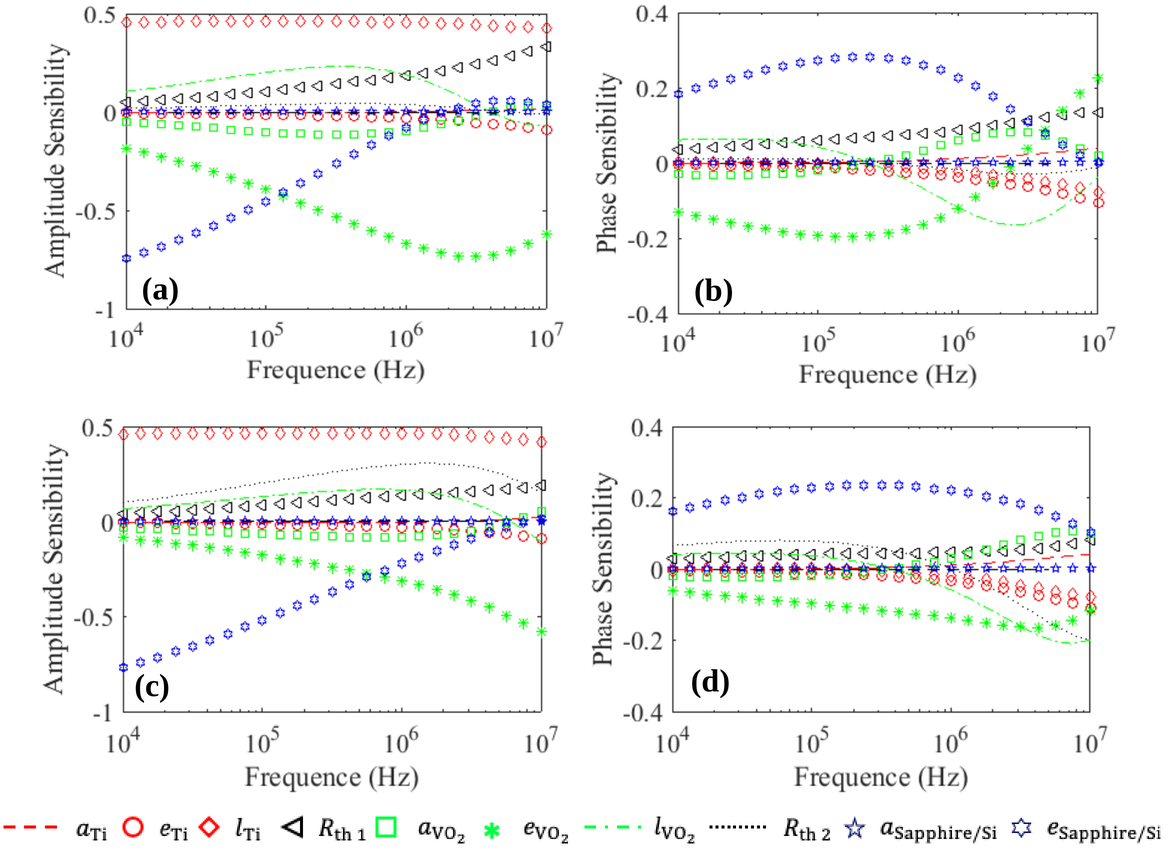

Figure S.4: Amplitude and phase sensitivities on all the experimental parameters using sample H.1(a,b) and sample H.2 (c,d) in the VO_2 insulator state at 28°C.

As can be seen in Figures S.4 and S.5, the PTR measurements are mainly sensitive to a_{VO_2} (green squares), e_{VO_2}(green asterisks) and $e_{sapphire}$ (blue hexagons). The influence of the Ti coating (red diamond and dashed line) is negligible compared to the substrates one (blue pentagram and hexagons). For that reason, the thermophysical properties of the Ti film and the substrate were taken from literature published values[7,8]. Additionally, the measurements are sensitive on both $R_{th\ 1}$and $R_{th\ 2}$ (resistance between VO_2 and the sapphire/Si substrates) at high temperatures (Figure S.5). Whereas, at low temperatures (Figure S.4) the experimental measurements are not sensitive to the second TBR ($R_{th\ 2}$) when using a sapphire substrate comparing to Si one.

These results lead to the conclusion that the nature of the substrate influences the sensitivities and therefore the extracted uncertainties of the parameter.

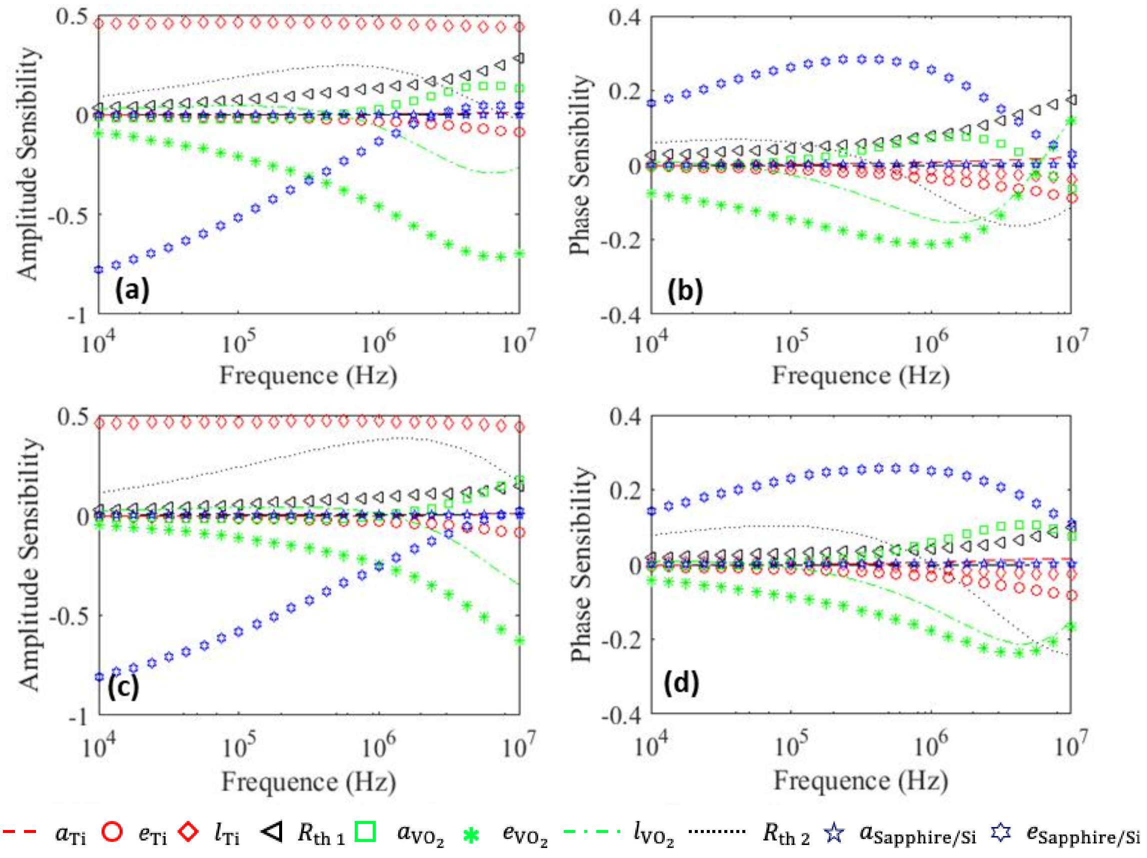

Figure S.5: Amplitude and phase sensitivities on all the experimental parameters using sample H.1(a,b) and sample H.2 (c,d) in the VO_2 metallic state at 78°C.

These sensitivities are then integrated into a least squares algorithm to compute the total uncertainty on the fitting parameters[9,10]. Equations S.2 and S.3 express two types of errors.

Where, σ_{residual} and $\sigma_{\text{input parameters}}$ are respectively the uncertainties related to the variance of the noise and that of the input parameters (i.e. supposed known parameters).

$$\sigma_{\text{residual}} = \sigma_{\text{noise}}[(X_r^t X_r)^{-1}]^{-1/2} \tag{S.2}$$

$$\sigma_{\text{input parameters}} = -[(X_r^t X_r)^{-1} X_r^t X_c \, e_{\beta c}] \tag{S.3}$$

σ_{noise} is the residual noise between the model and the experimental data; X_r is the sensitivity matrix of the fitting parameters; X_r^t is the transpose of X_r; X_c is the sensitivity matrix of the input parameter and $e_{\beta c}$ the uncertainty vector of the input parameters which are: a_{Ti}, e_{Ti}, l_{Ti}, $a_{\text{substrates}}$, $e_{\text{substrates}}$, l_{VO_2} $R_{\text{th 1}}$.

In order to combine these two uncertainties, a quadratic sum from the Guide to the Expression of Uncertainty in Measurement (GUM)[11] is used:

$$\Delta R_{th}^2 = 1.96^2\left(\sigma^2{}_{\text{residual}} + \sigma^2{}_{\text{input parameters}}\right) \tag{S.4}$$

The scaling factor 1.96 is given by the table of the Standard Normal Distribution for the repartition function on the detector opening for a confidence interval of 95%.

In the article, the errors of the measurements are given by the least square algorithm using the errors on the input parameters $e_{\beta c}$, taken from literature[7,8]. These errors were taken equal to: 5% on the thermophysical properties of Ti, sapphire, Si and on the thicknesses of the Ti and VO_2 layers; and 15% on $R_{\text{th 1}}$ (considering the assumption made).

References

1. Rogers, K. D. An X-ray diffraction study of semiconductor and metallic vanadium dioxide. *Powder Diffr.* **8**, 240–244 (1993).
2. Gomez-Heredia, C. L. *et al.* Thermal hysteresis measurement of the VO 2 emissivity and its application in thermal rectification. *Sci. Rep.* **8**, 8479 (2018).
3. Yang, T. H. *et al.* Semiconductor-metal transition characteristics of VO2thin films grown on c-and r-sapphire substrates. *J. Appl. Phys.* **107**, 0–6 (2010).
4. Weibel, A., Bouchet, R., Boulc', F. & Knauth, P. The big problem of small particles: a comparison of methods for determination of particle size in nanocrystalline anatase powders. *Chem. Mater.* **17**, 2378–2385 (2005).
5. Costescu, R., Wall, M. & Cahill, D. Thermal conductance of epitaxial interfaces. *Phys. Rev. B* **67**, 54302 (2003).
6. Gundrum, B. C., Cahill, D. G. & Averback, R. S. Thermal conductance of metal-metal interfaces. *Phys. Rev. B* **72**, 245426 (2005).
7. Touloukian, Y. S. & Buyco, E. H. *Specific Heat nonmetallic solids Volume 5*. (IFI/Plenum, 1970).
8. Touloukian, Y. S., Kirby, R. K., Taylor, R. E. & Desai, P. D. Volume 1 : Thermal conductivity - Metallic elements and alloys. *Thermophys. Prop. Matter-the TPRC Data Ser.* 1595 (1975).
9. Yang, J., Ziade, E. & Schmidt, A. J. Uncertainty analysis of thermoreflectance measurements. *Rev. Sci. Instrum.* **87**, 014901 (2016).
10. Y. Jarny, D. M. *Problèmes inverses et estimation de grandeurs en thermique, Métrologie thermique et techniques inverses*. (Cours C1A, Ecole d'Hiver METTI '99, Presses Universitaires de Perpignan, 1999).
11. Jcgm, J. C. F. G. I. M. Evaluation of measurement data — Guide to the expression of uncertainty in measurement. *Int. Organ. Stand. Geneva ISBN* **50**, 134 (2008).
